# Supplementary material for: Pretrained transformers applied to clinical studies improve predictions of treatment efficacy and associated biomarkers
Source: Nat Commun. 2025 Mar 1;16:2101. doi: 10.1038/s41467-025-57181-2 (PMC11873189; doi:10.1038/s41467-025-57181-2)
Supplement: Supplementary file 1 — Supplementary Information [file 41467_2025_57181_MOESM1_ESM.pdf]

Pretrained transformers applied to clinical studies improve predictions of treatment efficacy and associated biomarkers

Arango-Argoty G, Kipkogei E, Stewart R, Sun GJ, Patra A, Kagiampakis I, Jacob E

Supplementary Information

Supplementary Methods

Benchmarking against other neural network architectures

We evaluated the following neural-network approaches for survival prediction. We used default parameters for most of the architectures, except for Neural MTLR where the learning rate was set to 0.0001 because of exploding gradients, and for Transformer survival model where we selected the best model based on the plateau of the test loss:

- **Transformer Survival**[1]: Hu et al. proposed the use of the transformer architecture for survival analysis to estimates the patient's survival distribution. Input features are embedded using a feed forward network passed to a set of transformer encoder layers with a feed forward neural network as output to estimate the patient survival probabilities across a predefined time intervals. Parameters: default, 4 layers, 4 heads, 128 dim, 0.0001 learning rate.
- **Cox-nnet**[2]: Is a relatively simple neural network architecture composed of an input layer, one hidden layer of 143 neurons and an output layer of 1 node (a cox regression) with a tanh activation function. This model uses the partial log-likelihood as the cost function for optimizing the model's parameters. Parameters: default plus L2 regularization of 0.01
- **DeepSurv**[3]: is a flexible feed-forward neural network trained to estimate the log-risk function in the Cox model. The model is trained using the averaged negative log partial likelihood as the loss function. Parameters: default plus one layer with 128 neurons.
- **Neural MTLR**[4]: Is a neural Multi-Task Logistic Regression model that estimates the probability that an event occurs on a set of predefined time intervals. Parameters: default plus one layer with 128 neurons.
- **NNET-Survival**[5]: is a discrete-time survival model designed to predict the probabilities of failure (event) on a given time interval. The core of this model consists of a convolutional layer followed by a max pooling and a fully connected networks where the output layer dimension represents the time intervals, and the output scores the conditional log odds of surviving each time interval. The log odds is converted to conditional probabilities using a sigmoid activation function. Parameters: default parameters.

Data preprocessing

The Clinical Transformer was trained on a variety of datasets including different data modalities such as molecular (mutations, RNA seq), clinical, and demographic features. We performed different preprocessing steps depending on the data type as follows:

- **Mutational data**: We only considered missense and frameshift variants aggregated at the gene level by counting the number of mutations per gene.
- **Transcriptomics bulk RNA seq data**: We used gene expression data from TCGA to profile the tumor microenvironment in a pan cancer setting and specialized to melanoma samples. Training data (TCGA) as well as validation data (melanoma studies) were processed by extracting the 29 RNA signatures described in the Bagaev et al. study. In detail, all samples were log2(TPM+0.01) scaled and single sample gene set enrichment analysis (ssGSEA) was performed using R GSVA implementation. Thus, each patient was described by the 29 TME-related signatures.
- **Clinical and demographics data**: We did not perform any pre-processing to this data.

- **Data normalization**: Input data to the Clinical Transformer is scaled to the [0, 1] interval using the MinMaxScaler technique. All categorical variables were ordinally encoded.

All other modalities that did not need transformation (e.g., Chowell et al. dataset) were used exactly as they were provided by the publication where the data was downloaded.

Supplementary Note 1. Clinical Transformer training on the Chowell et al. dataset for comparison with Chowell's model.

To evaluate the Clinical Transformer' performance in the Chowell et al.[6] dataset, we used the same training/testing splits provided in their study. We pretrained a model using all the data in the training split for 20,000 epochs and stored all 20,000 model weights. We fine-tuned a Clinical Transformer specialized for predicting patient overall survival using a training split in which 90% of the training data was used to train the model and 10% was used for validation. To identify the best pretrained model for this task, we selected several pretrained snapshot models at 100, 500, 1000, 5000, 15,000, and 20,000 pretraining epochs. For each pretrained model we then fine-tuned a survival model. We selected the 20,000 pretrained model snapshot for transfer learning because it provided the best performance in the validation set. The best epoch was selected based on the average concordance index (C-index) across the 10 runs in the validation set. Once the best pretrained weights (20,000) and epoch (195) were defined from the validation split, we trained a new model using 100% of the training split for 195 epochs. We evaluated this model in the 20% test set and compared it against tumor mutation burden (TMB) and the random forest model from Chowell et al. study.

Supplementary Note 2. Evaluation of Clinical Transformer trained using the Chowell et al. dataset in the training/testing split framework and in the MYSTIC trial as independent dataset.

In this experiment, we merged the training and testing data from Chowell et al. into one single dataset and evaluated the performance of the Clinical Transformer against other survival techniques with and without pretraining, repeating the process 10 times. First, the data were divided into 80% for training and 20% for testing samples (10 times). Second, we pretrained the Clinical Transformer model using the entire dataset during 30,000 epochs. Third, we trained 10 Clinical Transformer models using the Chowell et al. training splits. Each fold was trained independently with and without the 30,000-pretraining snapshot. We empirically selected the best survival model epoch based on the mean C-index across 5% of the testing data, which was contrasted against the complete test set over the 10 splits (511 and 300 for the without and with pretraining models, respectively; MSI_SCORE and FCNA were unavailable in the MYSTIC trial [NCT02453282]). As the Clinical Transformer can handle missing features at inference, these two variables were not used in predicting patient survival scores.

The results showed that the model without pretraining achieved a C-index of 0.714, as compared with the model with pretraining, which had a C-index of 0.720; the random forest model, with a C-index of 0.714; Cox proportional hazards (PH), with a C-index of 0.709; and TMB, with a C-index of 0.55 (Supplementary Table 1).

We evaluated the performance of the Clinical Transformer using the Chowell et al. MYSTIC trial data by matching the features available on the trial. In total we identified a cohort of 150 patients (74 treated with PD-L1 and 76 with PD-L1 + CTLA-4) with a complete feature set (from 325 patients with available tissue TMB, only 150 had germline human leukocyte antigen [HLA] typing [HLA evolutionary divergence, HED]), except for MSI_SCORE and FCNA, which were unavailable in the clinical trial data. The 10 Clinical Transformer models trained on the Chowell

et al. data were evaluated on MYSTIC data. The model with pretraining achieved a C-index of 0.643, whereas the Clinical Transformer without pretraining obtained a C-index of 0.616 and TMB on MYSTIC data showed a C-index of 0.608. Random forest and the Cox PH model were not evaluated on the MYSTIC data, as those models cannot handle missing data. For patient stratification (Supplementary Figure 2), we extracted the median cutoffs from the training splits for the different methods (the direct and gradual learning as well as for the TMB score; (Supplementary Table 1, Supplementary Figure 2).

Supplementary Note 3. Samstein et al. dataset training/testing framework.

We pretrained the Clinical Transformer using all patient population from GENIE v11 using all available clinical and molecular data during 20,000 iterations.

We trained a Clinical Transformer model to predict patient overall survival in the Samstein et al. dataset, using 10 splits of 80% training and 20% testing. The baseline model corresponds to the model trained to predict survival without GENIE pretraining, and the E20000 model refers to the Clinical Transformer model trained on the Samstein et al. data, using the GENIE pretrained weights at the 20,000 snapshot.

Best epoch was empirically selected by looking at a small 5% proportion of the testing set in which 20% of the features were randomly shuffled to increase variability. We also evaluated the model's performance in the full testing set, using the average performance C-index across the 10 test sets. We did not find any significant difference in the testing at 5% and 100% evaluations. Therefore, we selected the models at the 25-training epoch (no difference in performance between epoch 23 to 30) for the pretrained model and the epoch 85 for the baseline model. Note that the same best epoch range (<25) was observed in an independent run using the Memorial Sloan Kettering Multimodal Integration of Dataset (80/20% data splits) with the GENIE pretrained at the 20,000 snapshot. Independently, these two runs confirmed that the best model using pretraining lay approximately at epoch 25.

Supplementary Note 4. Thorsson et al. dataset features.

The features reported by Thorsson et al.[7] characterized the tumor microenvironment (TME) using major immunogenomics methods for the assessment of total lymphocytic infiltrate (from genomic and hematoxylin-eosin image data), immune cell fractions from deconvolution analysis of mRNA sequencing data, immune gene expression signatures, neoantigen prediction, TCR and BCR repertoire inference, viral RNA expression, and somatic DNA alterations from TCGA.

Supplementary Note 5. Transfer learning: pretraining on GENIE and fine-tuning on smaller datasets.

We implemented pretraining and transfer learning using GENIE data and fine-tuned on small cohorts of patients to predict patient survival. To demonstrate the advantage of this approach, we implemented the following three stages: (1) pretraining, in which a model is trained on the GENIE v.11 dataset (N = 134,626), including 2,290 variables via standard masked self-supervised learning; (2) transfer learning, in which the GENIE model's weights are transferred to

a survival model with the same architecture but outputs a survival score; and (3) prediction of patient survival by fine-tuning the model on a small cohort of patients with a clear treatment line and survival endpoint. This third stage is benchmarked independently across four IO datasets: Samstein et al. pan-cancer (N = 1610),[8] lung cancer from MSK Multi-modal Integration of Data (MIND) (N = 246), the MYSTIC trial (N = 325), and the Dana Farber Cancer Institute melanoma dataset (N = 110) (Table 1). We evaluated direct and transfer learning models using the C-index and the number of iterations to reach peak performance over 10 training (80%) and testing (20%) splits (Supplementary Note 3).

Supplementary Note 6. Transfer learning: pretraining from the Chowell et al. dataset and transfer to MYSTIC.

We evaluated the added value of the transfer learning using the Chowell et al. dataset over the MYSTIC dataset. First, we pretrained a model using all the data from the Chowell et al. dataset (train + test splits) and used the model snapshot at epoch 30,000. The pretrained model weights were transferred to a specialized model to predict patient overall survival using the MYSTIC data. For survival analysis, the MYSTIC data were divided into 10 training and testing splits, and 10 models were trained with and without pretraining. We selected the best model based on the averaged C-index of the 10 models in the test set. A random survival forest, a Cox PH model, and TMB were used for comparison (Table 2).

Supplementary Note 7. Foundation model: pretraining with GENIE and fine-tuning on the MSK-MeTropism dataset.

We trained the Clinical Transformer on GENIE v15 data (excluding samples from MSK-MeTropism; 167,421 samples) for 10,000 iterations, and fine-tuned it on MSK-MeTropism prostate cancer data (1,762 samples from metastatic disease: 939 primary tumor samples and 823 metastatic tumor samples). We did not use our pre-trained GENIE v11 foundation model, as it already included sample type as an input feature and MSK-MeTropism samples, precluding its use for this task. Training included all available molecular features aggregated at the gene level (missense and frameshift variants only, with copy number variants pre- extracted at the gene level) alongside patient age, sex, race, cancer type, sample type, cancer type detailed, sample type detailed, and sequencing center.

The Clinical Transformer with fine-tuning outperformed the random forest model on the average test set AUROC across all 10 times repeated train/test split data fractions (Supplementary Table 3). The advantage was most pronounced with small training sizes; using only 5% of data for training, the Clinical Transformer achieved an AUC of 0.761 (±0.011) compared to 0.722 (±0.017) for random forest.

Supplementary Note 8. Transfer learning with GENIE to the Samstein et al. dataset using only mutational data.

We trained a Clinical Transformer model using only the mutational data from the Samstein et al. dataset (469 gene mutations) without any other clinical or aggregated feature (e.g., race, age, TMB). This experiment was conducted to test the ability of the Clinical Transformer to identify

molecular features associated with patient survival while being unbiased to any aggregated feature such as TMB. We used the pretrained GENIE dataset at 20,000 epochs and trained the survival model for 10 repetitions, using splits of 80% training and 20% testing. The best model epoch was defined at 25 and was empirically selected as the average of the 10 model's performance in the test set. We evaluated the performance of the model using C-index. Note that the model trained only with mutational data (20,000 epochs, E020000) underperformed compared with the model that included clinical data (20,000 epochs, E020000B) but still outperformed the baseline without pretraining. For model interpretability, we computed all pairs cosine-similarities over the 10 test sets for consistency and extracted 50 functional groups by using a hierarchical clustering algorithm (Supplementary Figure 5). These functional groups represent the molecular associations within the data and their impact on survival.

Supplementary Note 9. Variant/invariant populations in the Chowell et al. dataset.

Probabilistic impact on patient survival is measured by the standard deviation of the distribution of all survival scores when one or two variables are perturbed. If a patient has a high standard deviation, it reflects changes in survival scores, whereas patients with standard deviation close to 0 indicate that the patient is not sensitive to perturbations in the given variable(s).

Supplementary Note 10. Positional encoding impact on model predictions.

To confirm that the Clinical Transformer, which lacks position encoding, is not sensitive to feature order, we performed the following experiment. We trained a modified version of a Clinical Transformer model on data from Chowell et al. that lacks feature order shuffling (i.e. samples features in a constant order). During model inference, we shuffled the order of features and generated 10 shuffled replicates for each sample. We then computed the standard deviation of model predictions for each sample against their 10 replicates. The standard deviations showed an exceedingly low variance (below 1e-6; Supplementary Figure 10). This result provides clear empirical evidence that feature order does not affect the predictive consistency of the Clinical Transformer.

Supplementary Note 11. Choosing the number of clusters (functional groups).

For the identification of the number of functional groups from the cosine similarity scores, we used a conventional silhouette score-based methodology (arbitrary, other method could have been chosen). For instance, in the Chowell et al., dataset, we used the k-means clustering algorithm across a predefined range of potential cluster numbers (2 to 10). The silhouette score for each predefined number of clusters was computed to quantitatively assess the clustering quality. This metric evaluates the degree of similarity of an instance to its assigned cluster in relation to other clusters. The selection of the optimal number of clusters was defined by the highest silhouette score (in this case k=4). This approach ensures that the chosen cluster solution maximizes intra-cluster similarity while maintaining clear delineation between different clusters.

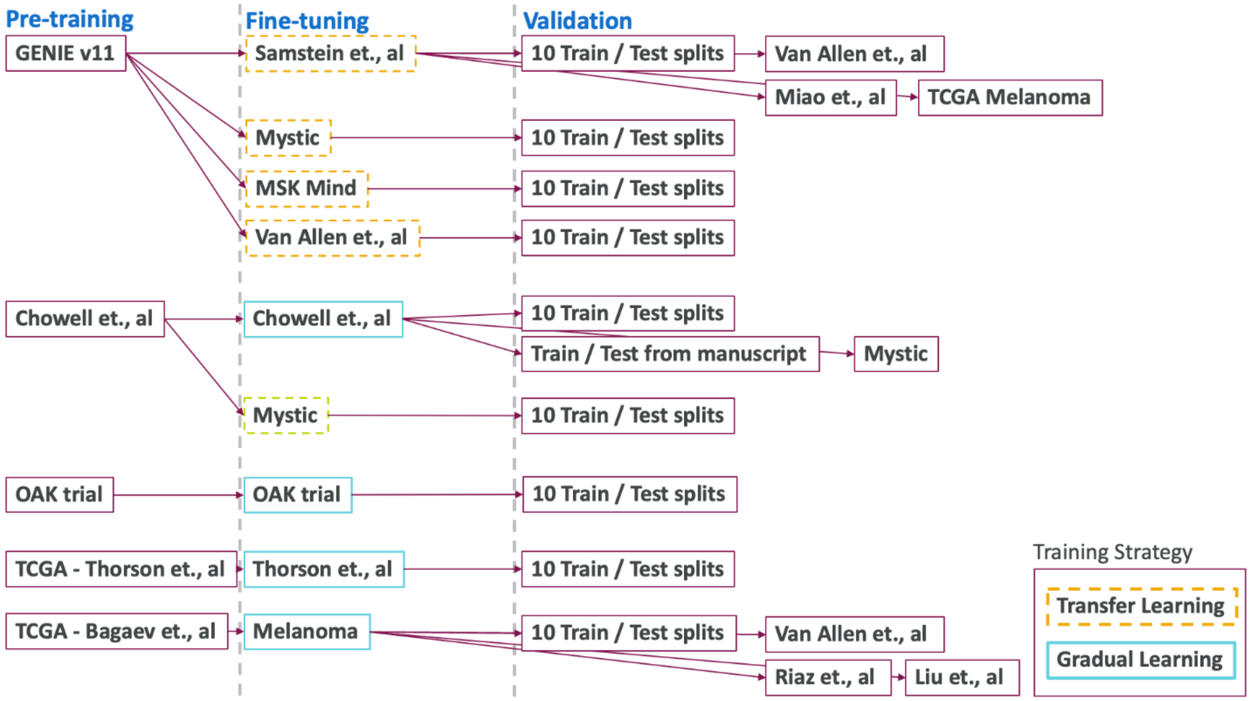

Supplementary Figure 1. Experimental data flow for Clinical Transformer.

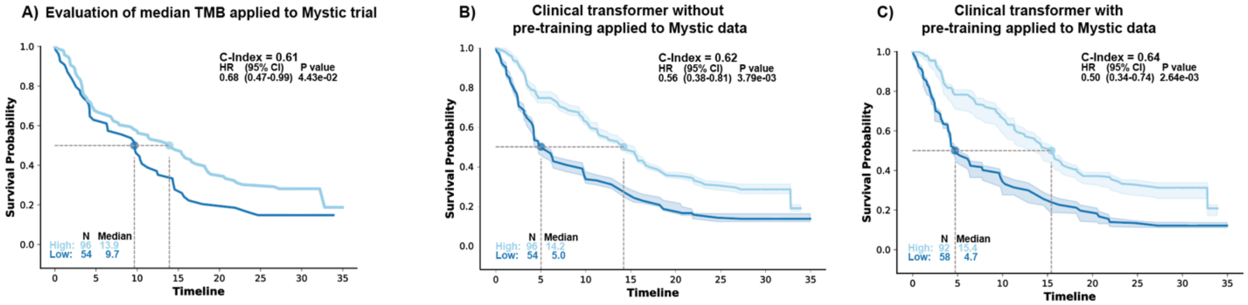

Supplementary Figure 2. Patient stratification using **a**, TMB; **b**, direct; and **c**, gradual learning in the test sets (10 repetitions). Patient population was stratified using the median cutoff from the training splits. Solid line indicates the averaged KM curve across the 10 training repetitions and the areas represent the variability across the 10 testing splits. P-values for hazard ratios were computed with a Wald test. Source data are provided in the SupplementarySourceData file.

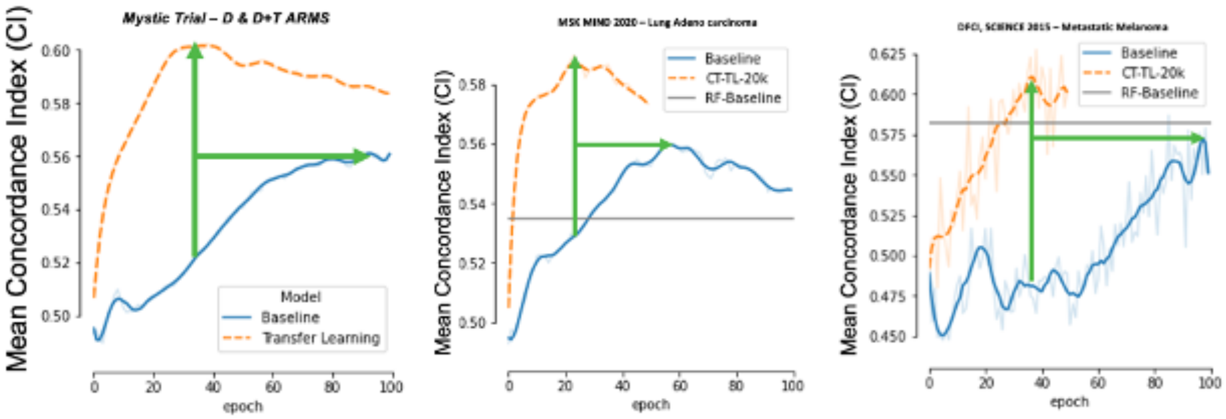

Supplementary Figure 3. Positive effect of transfer learning from pretrained model using GENIE to other small datasets. Models pretrained with GENIE dataset achieved a peak performance in a smaller number of epochs compared with baseline Clinical Transformer trained models. Source data are provided in the SupplementarySourceData file.

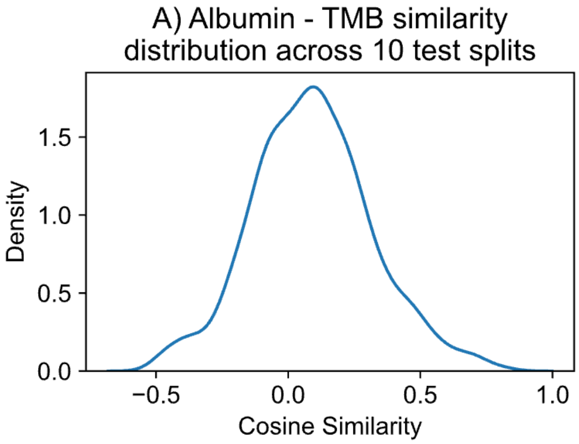

Supplementary Figure 4. Albumin–TMB cosine similarity score across the test populations in Chowell et al.[6] dataset. Cosine similarity distribution.

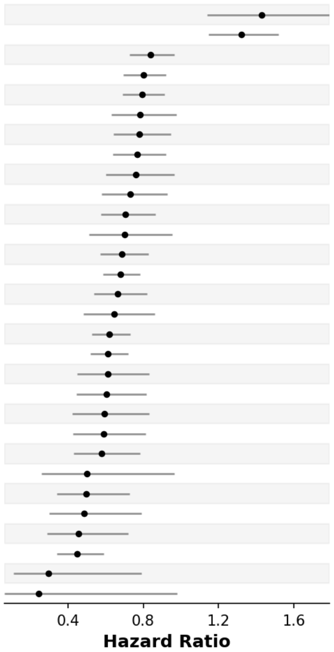

Variable	(95% Conf Int)	Pval	Functional Groups
C48	1.43(1.14 to 1.79)	0.0***	KEAP1 STK11
C47	1.32(1.15 to 1.52)	0.0***	APC PIK3CA TP53
C28	0.84(0.73 to 0.96)	0.01**	ALK ARID2 BRD4 CREBBP DOT1L EPHA5 EPHA7 ERBB4 FAT1 FLT4
C3	0.80(0.70 to 0.92)	0.0***	ASXL1 ATM ATRX BRAF CTNNB1 EGFR EP300 ERBB2 FBXW7 KMT2C
C14	0.79(0.69 to 0.91)	0.0***	AMER1 AR ARID5B ASXL2 ATR AXIN1 AXIN2 BLM BRCA1 CDK12
C34	0.78(0.63 to 0.98)	0.03**	E2F3 FGF3 IRF4 MAP2K2 MYOD1 NEGR1 PIK3C3 PTPN11 RASA1 SDHA
C24	0.78(0.64 to 0.95)	0.01**	BAP1 BIRC3 CD79B FYN GATA1 INPP4A KLF4 MST1R NKX2-1 PLK2
C16	0.77(0.64 to 0.92)	0.0***	ARAF ETV1 EZH2 IFNGR1 KIT MAP3K1 NF2 PRKAR1A RAF1 REL
C4	0.76(0.60 to 0.96)	0.02**	B2M CASP8 KMT2B MSH3 PPP2R1A SMAD4 TCF7L2
C19	0.73(0.58 to 0.93)	0.01***	FGFR3 PIK3R1 PPM1D RUNX1 TSC1
C11	0.71(0.58 to 0.87)	0.0***	CCND2 CDK6 CHEK2 CXCR4 EIF1AX FAM175A FANCC FGF19 FGFR1 FOXO1
C5	0.70(0.51 to 0.96)	0.02**	BABAM1 BCL2 CCND1 ELF3 ERF HIST1H2BD HIST1H3I MAPK1 MAPKAP1 MAX
C32	0.69(0.57 to 0.83)	0.0***	DDR2 DNMT3B ERCC2 ERCC3 ERCC5 ETV6 FGFR4 FOXP1 JAK3 LATS1
C35	0.68(0.59 to 0.78)	0.0***	ANKRD11 ARID1B BCOR BRCA2 CARD11 CIC MSH6 NOTCH3 NOTCH4 PAK7
C10	0.67(0.54 to 0.82)	0.0***	BCL2L11 FH FLCN FUBP1 HIST1H3B HOXB13 JAK1 NCOA3 NUP93 RAD54L
C17	0.64(0.48 to 0.86)	0.0***	AKT3 BBC3 CEBPA CUL3 ERRFI1 GNA11 RAD21 RIT1 SHQ1 SMARCB1
C7	0.62(0.53 to 0.73)	0.0***	CBL CSF3R CTCF DNMT3A ERBB3 ESR1 FGFR2 GATA3 HNF1A INPP4B
C23	0.61(0.52 to 0.72)	0.0***	ALOX12B AXL BARD1 BRIP1 CSF1R DIS3 ERCC4 ERG FANCA FLT3
C6	0.61(0.45 to 0.83)	0.0***	AURKA EIF4A2 GATA2 GPS2 GSK3B IGF2 JUN RAD51B SUZ12
C9	0.60(0.45 to 0.82)	0.0***	CALR CDKN1B CTLA4 CYLD DROSHA EPAS1 EPCAM MAPK3 SMAD2 SOCS1
C13	0.59(0.42 to 0.83)	0.0***	CDKN1A CRLF2 DNAJB1 EED PPARG RAC1
C38	0.59(0.43 to 0.81)	0.0***	ABL1 BCL6 CHEK1 MUTYH PIK3R2 RARA SUFU
C8	0.58(0.43 to 0.78)	0.0***	AKT2 BTK CDC73 HLA-B IKBKE INPPL1 RFWD2 TRAF2 WHSC1
C12	0.50(0.26 to 0.96)	0.04**	CSDE1 FAM58A NTHL1 PRKCI SESN3 SH2D1A
C18	0.50(0.34 to 0.73)	0.0***	BMPR1A CDK4 MAP2K1 MSI2 NKX3-1 PPP6C WHSC1L1
C15	0.49(0.30 to 0.79)	0.0***	CD79A FAM46C HIST1H3C NPM1 SRSF2 VEGFA
C22	0.46(0.29 to 0.72)	0.0***	CCNE1 CD274 FOXL2 PAK1 RAD51C RYBP SDHAF2
C0	0.45(0.34 to 0.59)	0.0***	IDH1 VHL
C33	0.30(0.11 to 0.79)	0.02**	DUSP4 KNSTRN LYN MSI1 SHOC2 STK19 TEK
C45	0.24(0.06 to 0.98)	0.05**	MST1

Supplementary Figure 5. CoxPH HR (error bars: 95% confidence interval) of significant ($P < 0.05$) functional groups, as computed over Samstein et al.[8] dataset, comparing patients with at least 1 mutated gene in the given functional group vs. those with no mutated genes in the given functional group. P-values for hazard ratios were computed with a Wald test. Conf Int, confidence interval; Pval, p-value. Source data are provided in the SupplementarySourceData file.

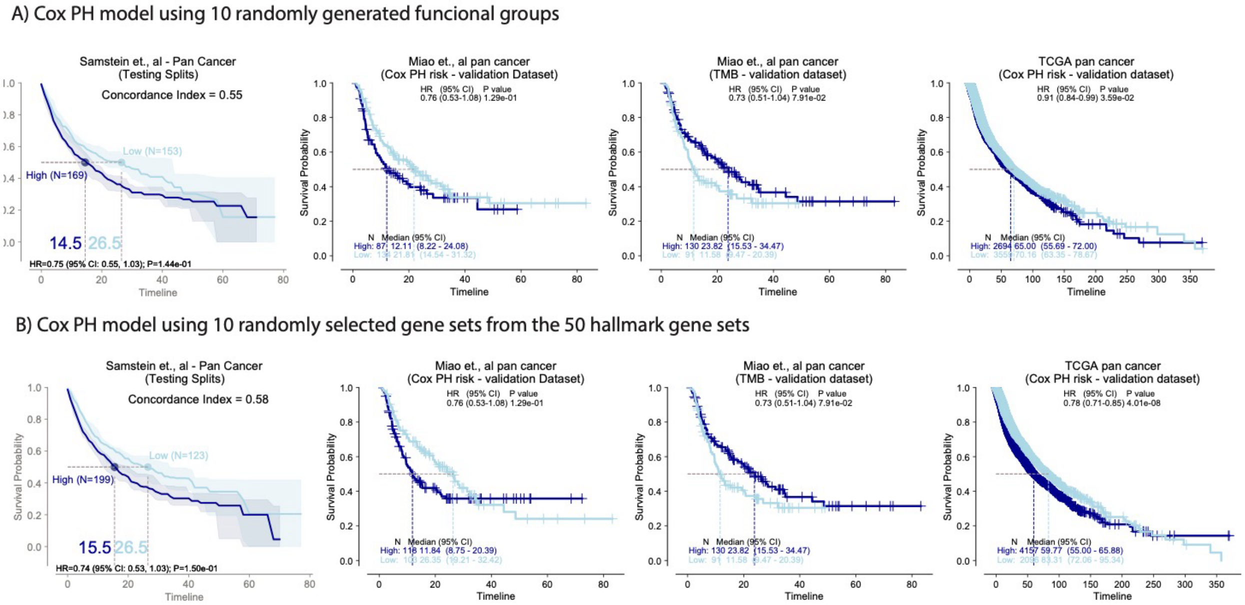

Supplementary Figure 6. Integration of functional groups to predict patient survival with immuno-oncology (IO) treatment in the testing splits (discovery dataset) as well as on Miao et al.[9] pan-cancer IO-treated patients and treatment-naive pan-cancer datasets in The Cancer Genome Atlas (TCGA). **a**, Evaluation of randomly generated functional groups of the same size and number of genes as the top 10 groups. **b**, Evaluation of 10 randomly selected hallmark gene sets from the complete 50 hallmark sets and subsetting it to the MSK panel genes. P-values for hazard ratios were computed with a Wald test. Source data are provided in the SupplementarySourceData file.

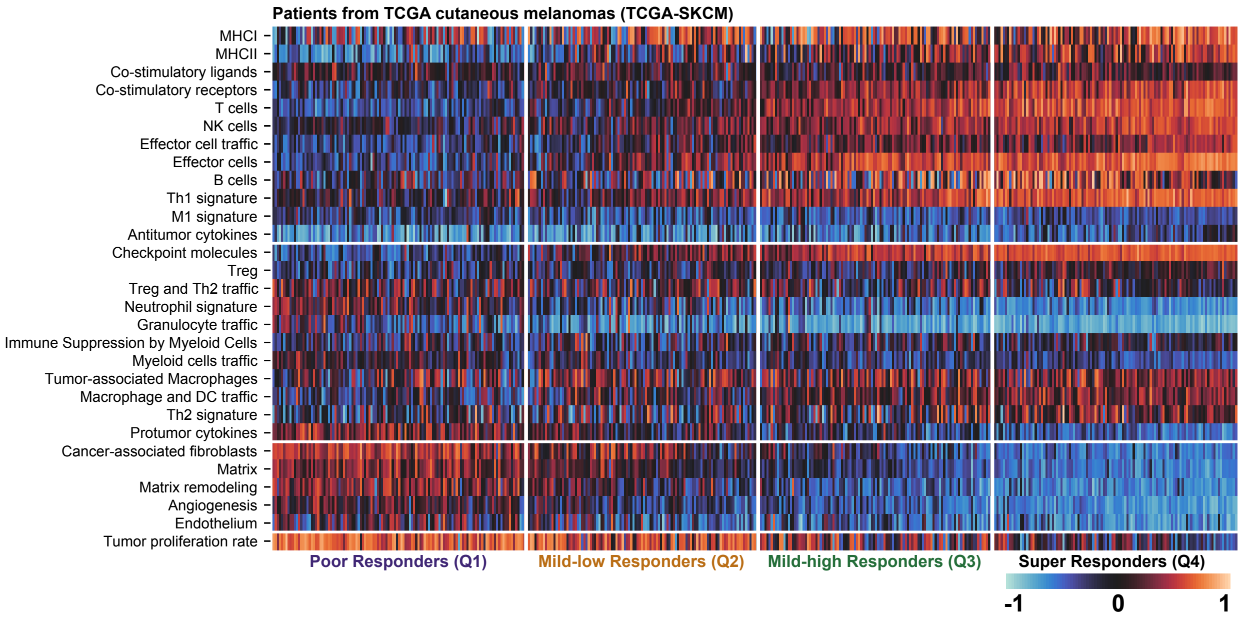

Supplementary Figure 7. Distribution of gene expression signatures across the four survival groups. Groups Q3 and Q4, with prolonged survival, demonstrated an increase in expression of signatures associated with productive antitumor immunity, such as major histocompatibility complex, T-cells, and T helper type 1 cell signaling. In contrast, groups Q1 and Q2, with reduced survival, demonstrate reduced expression of these signatures commensurate with an increase in signatures of neutrophils, protumor inflammatory signals, cancer-associated fibroblasts, and matrix remodeling. Source data are provided in the SupplementarySourceData file.

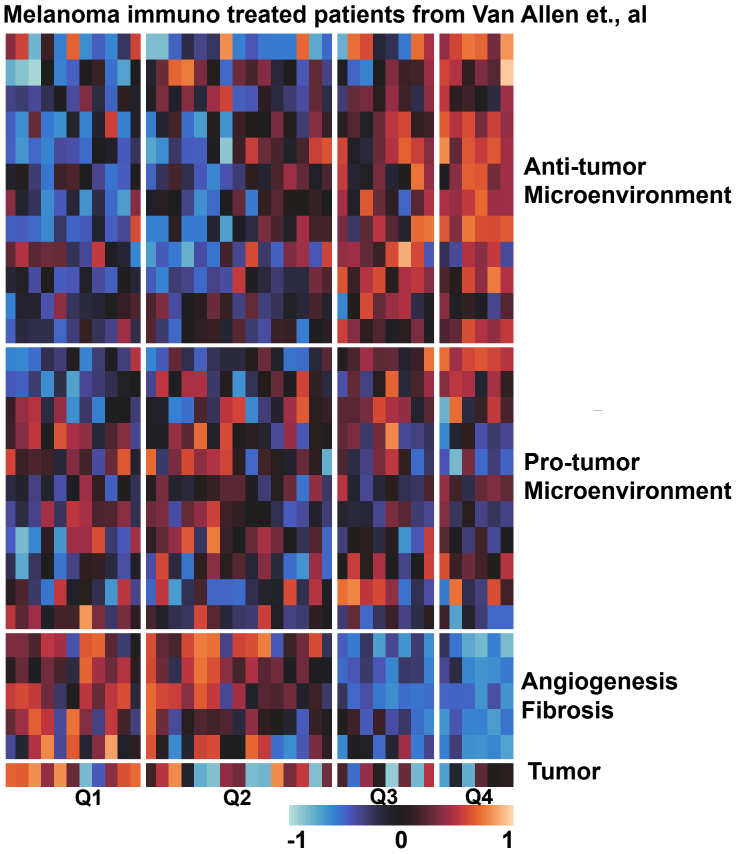

Supplementary Figure 8. Heat map of the tumor microenvironment signatures in the Van Allen et al. dataset.[10] Source data are provided in the SupplementarySourceData file.

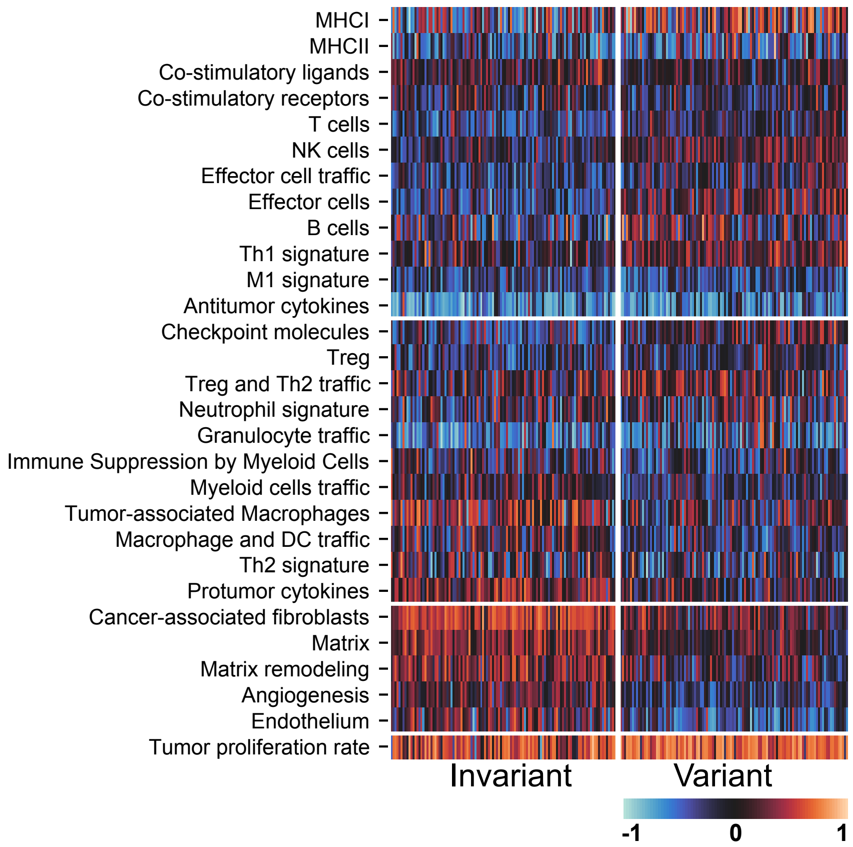

Supplementary Figure 9. Heat map of tumor microenvironment enrichment for the invariant (left column) and variant (right column) populations within TCGA-SKCM. Source data are provided in the SupplementarySourceData file.

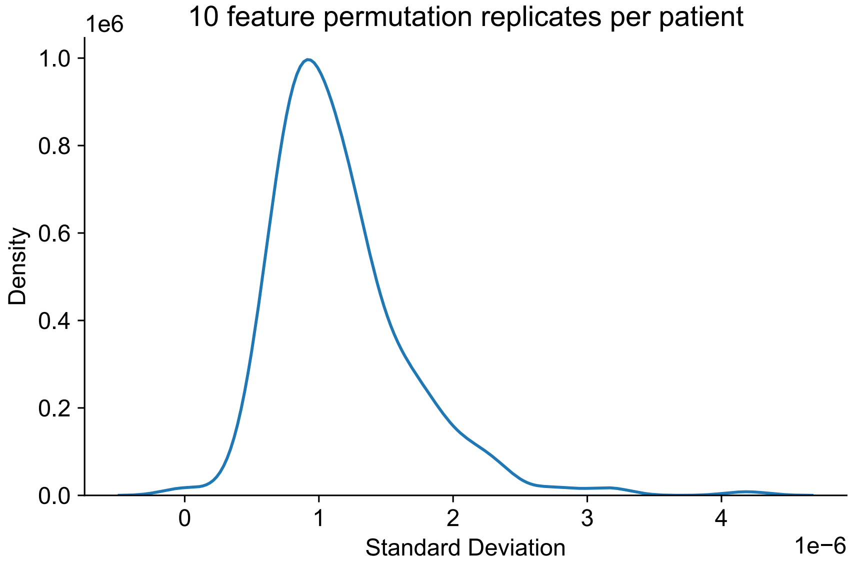

Supplementary Figure 10. Standard deviation of survival score across 10 feature-permuted replicates for each patient. SuSource data are provided in the SupplementarySourceData file.

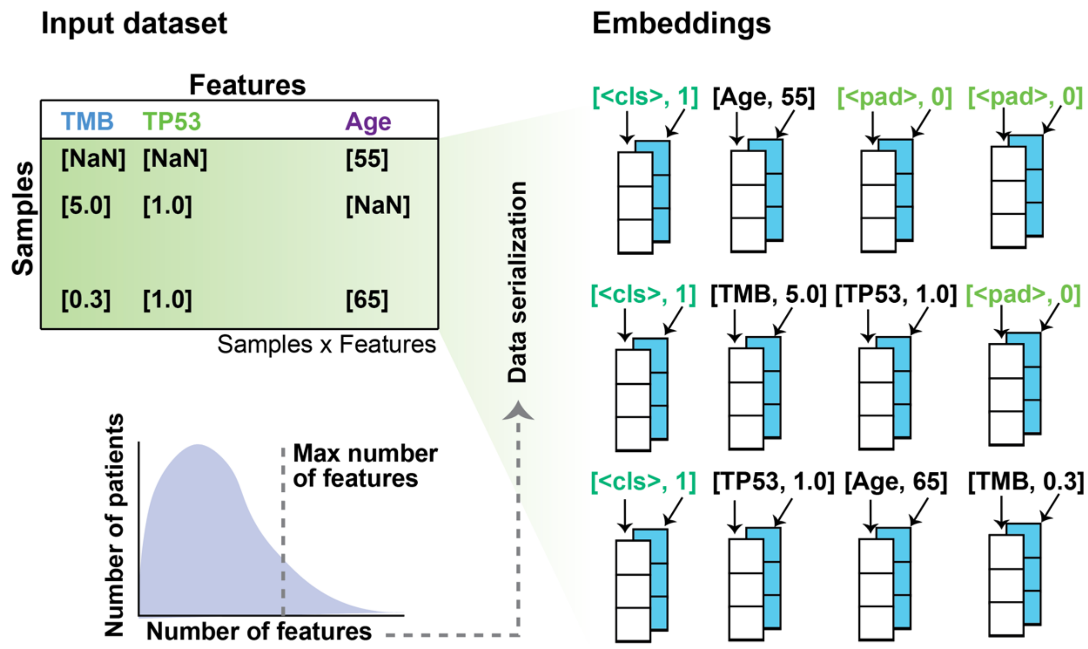

Supplementary Figure 11. Input data processing flow for Clinical Transformer.

Supplementary Table 1. Performance of the Clinical Transformer model trained on the Chowell et al. dataset and evaluated on the MYSTIC trial. Results reported as mean c-index ± standard deviation across 10 train/test splits. Boldface indicates modeling framework with best performance.

Modeling framework		Chowell et al. 2021	MYSTIC (validation)
Clinical Transformer	Direct learning	0.714 ± 0.01	0.616 ± 0.004
	Gradual learning	**0.720 ± 0.01**	**0.643 ± 0.004**
Linear modeling	Cox PH regression	0.709 ± 0.01	—
Nonlinear modeling	Random survival forest	0.714 ± 0.01	—
Biomarkers	TMB	0.550 ± 0.02	0.608 ± 0.000

Supplementary Table 2. Impact of GENIE transfer learning. The GENIE pretrained model was used to fine-tune survival time and event across four datasets. **Top:** C-index of best model (mean ± standard deviation across 10 train/test splits) for both direct and gradual learning. **Bottom**: Number of epochs (or iterations) the model needs to achieve peak performance. Column headers in bold.

**Learning type**	**Samstein et al.[8]**	**DFCI melanoma**	**MYSTIC trial**	**MSK MIND**
**Concordance index**				
Direct learning	0.627 ± 0.02	0.587 ± 0.10	0.561 ± 0.05	0.560 ± 0.02
Transfer learning	0.649 ± 0.02	0.628 ± 0.07	0.602 ± 0.05	0.590 ± 0.03
**Training epochs**				
Direct learning	86	85	95	57
Transfer learning	27	38	38	23
Average reduction (%)				39.1130271

Supplementary Table 3. Model performance for the metastatic or primary prediction task at different training sizes. AUC mean scores and standard deviations over 10 training / testing splits. Boldface indicates model with best performance. TL1, Transfer learning from GENIE using the pre-trained 100 epoch snapshot; TL2, Transfer learning from GENIE using the pre-trained 10,000 epoch snapshot; RF, Random forest model.

	AUC @ Training data (%)										
Model	1	5	10	20	30	40	50	60	70	80	90
TL1	**0.690 (±0.045)**	**0.761 (±0.011)**	**0.770 (±0.008)**	**0.776 (±0.004)**	**0.781 (±0.006)**	**0.784 (±0.010)**	**0.786 (±0.017)**	**0.788 (±0.012)**	**0.788 (±0.022)**	**0.790 (±0.024)**	**0.799 (±0.026)**
TL2	0.673 (±0.068)	0.756 (±0.010)	0.766 (±0.010)	0.771 (±0.008)	0.774 (±0.009)	0.776 (±0.012)	0.779 (±0.019)	0.782 (±0.016)	0.782 (±0.024)	0.784 (±0.022)	0.792 (±0.025)
RF	0.679 (±0.057)	0.722 (±0.037)	0.738 (±0.017)	0.757 (±0.013)	0.767 (±0.011)	0.768 (±0.010)	0.773 (±0.010)	0.783 (±0.016)	0.784 (±0.017)	0.784 (±0.019)	0.784 (±0.025)

Supplementary Table 4. Pairwise cosine similarity between all feature embeddings across 10 test splits in Chowell et al.[6] dataset. Column headers in bold.

**source**	**target**	**cosine**	**cluster**
Albumin	TMB	0.090804	-1
Albumin	HLA_LOH	0.102075	-1
MSI_SCORE	Stage at IO start	0.12652	-1
Cancer_Type	MSI_SCORE	0.130802	-1
Drug_class	MSI_SCORE	0.132756	-1
Cancer_Type	TMB	0.14081	-1
Chemo_before_IO (1:Yes; 0:No)	HLA_LOH	0.142047	-1
Drug_class	TMB	0.152745	-1
Chemo_before_IO (1:Yes; 0:No)	TMB	0.15311	-1
Stage at IO start	TMB	0.153569	-1
Age	HLA_LOH	0.157596	-1
Platelets	TMB	0.163091	-1
Cancer_Type	HLA_LOH	0.167021	-1
Drug_class	HLA_LOH	0.16719	-1
HLA_LOH	Stage at IO start	0.16879	-1
Cancer_Type	HED	0.173664	-1
Drug_class	HED	0.179617	-1
HED	Stage at IO start	0.179697	-1
HLA_LOH	Platelets	0.180411	-1
Age	TMB	0.180579	-1
HGB	Stage at IO start	0.193215	-1
Albumin	Drug_class	0.193443	-1
Albumin	Stage at IO start	0.200334	-1
Drug_class	HGB	0.201872	-1
HGB	HLA_LOH	0.203599	-1
Cancer_Type	HGB	0.205256	-1
HGB	TMB	0.209394	-1
Cancer_Type	NLR	0.210308	-1
Albumin	Cancer_Type	0.213965	-1

source	target	cosine	cluster
NLR	Stage at IO start	0.216547	-1
Drug_class	NLR	0.217594	-1
MSI_SCORE	Platelets	0.22695	-1
BMI	HLA_LOH	0.22727	-1
Chemo_before_IO (1:Yes; 0:No)	MSI_SCORE	0.232161	-1
BMI	Stage at IO start	0.23337	-1
BMI	Cancer_Type	0.235731	-1
HLA_LOH	Sex (1:Male; 0:Female)	0.239134	-1
FCNA	HLA_LOH	0.245705	-1
HED	Platelets	0.245854	0
BMI	Drug_class	0.246851	-1
Albumin	MSI_SCORE	0.252161	-1
FCNA	TMB	0.258304	-1
BMI	TMB	0.259914	-1
Age	MSI_SCORE	0.269567	-1
FCNA	MSI_SCORE	0.277269	-1
Platelets	Stage at IO start	0.286154	-1
Age	Stage at IO start	0.291318	-1
Cancer_Type	Platelets	0.293372	-1
Drug_class	Platelets	0.294873	-1
Chemo_before_IO (1:Yes; 0:No)	HED	0.298438	-1
Age	Drug_class	0.302489	-1
Age	Cancer_Type	0.303002	-1
Sex (1:Male; 0:Female)	TMB	0.307323	-1
MSI_SCORE	Sex (1:Male; 0:Female)	0.311496	-1
HGB	Sex (1:Male; 0:Female)	0.312721	-1
Chemo_before_IO (1:Yes; 0:No)	NLR	0.322467	-1
HED	HLA_LOH	0.322497	-1
HGB	MSI_SCORE	0.325534	-1
BMI	MSI_SCORE	0.328931	-1

source	target	cosine	cluster
Chemo_before_IO (1:Yes; 0:No)	Platelets	0.347991	-1
Chemo_before_IO (1:Yes; 0:No)	HGB	0.353559	-1
NLR	Platelets	0.355904	0
FCNA	HED	0.357096	-1
Age	FCNA	0.360321	-1
Age	Platelets	0.367669	0
Platelets	Sex (1:Male; 0:Female)	0.368122	-1
Age	NLR	0.380484	0
FCNA	NLR	0.382884	-1
Albumin	HED	0.386779	0
HLA_LOH	NLR	0.388365	-1
FCNA	HGB	0.390842	-1
HED	MSI_SCORE	0.39182	-1
Age	Chemo_before_IO (1:Yes; 0:No)	0.394198	-1
HED	Sex (1:Male; 0:Female)	0.396839	-1
BMI	Sex (1:Male; 0:Female)	0.401142	-1
HED	NLR	0.413955	0
BMI	Chemo_before_IO (1:Yes; 0:No)	0.422232	-1
NLR	Sex (1:Male; 0:Female)	0.42726	-1
BMI	HED	0.428204	0
Age	Sex (1:Male; 0:Female)	0.43022	-1
NLR	TMB	0.431291	-1
HED	TMB	0.43216	-1
Albumin	Chemo_before_IO (1:Yes; 0:No)	0.444123	-1
Age	HED	0.448813	0
HGB	Platelets	0.449957	0
Albumin	FCNA	0.45593	-1
Albumin	Sex (1:Male; 0:Female)	0.457991	-1
BMI	FCNA	0.459169	-1

source	target	cosine	cluster
BMI	NLR	0.459716	0
Cancer_Type	Chemo_before_IO (1:Yes; 0:No)	0.459975	-1
Chemo_before_IO (1:Yes; 0:No)	Stage at IO start	0.463177	-1
Age	Albumin	0.468315	0
HGB	NLR	0.46935	0
Albumin	BMI	0.471736	0
Chemo_before_IO (1:Yes; 0:No)	Drug_class	0.478527	-1
MSI_SCORE	NLR	0.482745	-1
Albumin	NLR	0.490508	0
HED	HGB	0.49444	0
FCNA	Platelets	0.512317	-1
Chemo_before_IO (1:Yes; 0:No)	FCNA	0.536875	3
Age	HGB	0.5395	0
Cancer_Type	FCNA	0.541182	-1
Cancer_Type	Sex (1:Male; 0:Female)	0.547098	-1
Drug_class	FCNA	0.550968	-1
Albumin	Platelets	0.556542	0
BMI	HGB	0.558014	0
Drug_class	Sex (1:Male; 0:Female)	0.558315	-1
Sex (1:Male; 0:Female)	Stage at IO start	0.560583	-1
FCNA	Stage at IO start	0.578723	-1
Age	BMI	0.592643	0
MSI_SCORE	TMB	0.59388	2
BMI	Platelets	0.60135	0
Albumin	HGB	0.606935	0
Chemo_before_IO (1:Yes; 0:No)	Sex (1:Male; 0:Female)	0.622801	3
FCNA	Sex (1:Male; 0:Female)	0.66105	3
HLA_LOH	MSI_SCORE	0.675359	2
HLA_LOH	TMB	0.696034	2
Cancer_Type	Drug_class	0.972104	1

source	target	cosine	cluster
Cancer_Type	Stage at IO start	0.97506	1
Drug_class	Stage at IO start	0.980401	1

Supplementary Table 5. Mean value of tumor microenvironment gene signatures (feature; Bagaev et al.[11]) computed for each of the patient subgroups that changed (mean variant) or did not change (mean invariant) upon perturbation of the T-cell gene expression signature within the Q1 and Q2 patients of the TCGA-SKCM cohort. Signatures are sorted by the max difference (delta) between variant and invariant subgroups. P-value (pval) is reported from a Bonferroni-corrected two-sided Mann-Whitney-Wilcoxon test comparing the distributions of variant and invariant patient populations. Column headers in bold.

**Feature**	**pval**	**Mean variant**	**Mean invariant**	**delta**
Endothelium	1.521E-15	-0.313412674	0.112600806	-0.426013479
Cancer-associated fibroblasts	4.069E-20	0.128167853	0.498169156	-0.370001303
Angiogenesis	2.233E-15	-0.200509689	0.120291558	-0.320801246
Matrix remodeling	1.422E-10	-0.014565958	0.28148602	-0.296051978
Matrix	1.543E-14	0.057901648	0.331919433	-0.274017785
Protumor cytokines	8.999E-11	-0.000761741	0.269065383	-0.269827124
Tumor-associated Macrophages	2.992E-06	-0.015938159	0.221935134	-0.237873292
Macrophage and DC traffic	5.583E-06	-0.189935965	0.02157267	-0.211508635
Myeloid cells traffic	2.537E-07	-0.164258572	0.025172696	-0.189431268
Th2 signature	0.005889	-0.160534751	-0.010702139	-0.149832612
Immune Suppression by Myeloid Cells	0.009670	-0.176916701	-0.060527783	-0.116388918
Co-stimulatory ligands	0.04121	0.013129773	0.093677403	-0.08054763
Antitumor cytokines	0.03496	-0.486369988	-0.567646764	0.081276776
Treg and Th2 traffic	0.02423	0.137531893	0.032257012	0.105274881
Tumor proliferation rate	1.820E-06	0.632009267	0.458450045	0.173559222
B cells	0.0003497	0.054694791	-0.132986461	0.187681252
Th1 signature	5.721E-07	0.18682555	-0.001916572	0.188742123
T cells	1.123E-07	-0.081965987	-0.280401733	0.198435746
Effector cell traffic	2.823E-06	-0.044440517	-0.247127222	0.202686705
NK cells	3.487E-12	0.150490246	-0.115612173	0.266102419
Checkpoint molecules	3.976E-10	0.053729001	-0.233654563	0.287383564
MHCI	7.635E-06	0.213198128	-0.091958146	0.305156274
Effector cells	2.997E-16	0.130232828	-0.271111321	0.401344149

Supplementary Table 6. CoxPH hazard ratios (hr) between populations when stratifying a cohort (cohort) on the global median (computed across Allen,[10] Liu,[12] and Riaz[13] studies; TCGA[11] cohort used its own median to stratify) of a given TME gene signature (var; subset of signatures from Bagaev et al.[11] that were either the top four with the largest magnitude of difference between variant and invariant populations, or the bottom four with the most negative difference between variant and invariant populations; a given signature level was equal to the mean expression across all genes within that signature; see also Supplementary Table 5). hr, HR when comparing the subpopulation with its gene signature ≤ the global median for that gene signature versus the subpopulation > the global median for that gene signature; hr_lower95, the lower bound of the 95% confidence interval for the HR point estimate; hr_upper95, the upper bound of the 95% confidence interval for the HR point estimate; N_high, number of patients in the subpopulation with its gene signature > the global median for that gene signature; N_Low, number of patients in the subpopulation with its gene signature ≤ the global median for that gene signature. P-values for hazard ratios were computed with a Wald test. Column headers in bold.

**Population**	**hr**	**hr_lower95**	**hr_upper95**	**pval***	**n_low**	**n_high**	**cohort**	**var**
IO	0.53141082	0.40298061	0.70077183	7.50E-06	227	227	tcga	Effector cells
IO	0.53032084	0.24673068	1.13986711	0.10423856	18	22	allen	Effector cells
IO	0.53338112	0.25117768	1.13264609	0.10188323	18	24	liu	Effector cells
IO	0.35451605	0.12128437	1.03625577	0.05810732	14	12	riaz	Effector cells
IO	0.85388965	0.64953366	1.12254003	0.25774627	227	227	tcga	MHCI
IO	0.76860871	0.3553994	1.66224068	0.50367427	23	17	allen	MHCI
IO	0.65615835	0.31400182	1.37115058	0.26248648	18	24	liu	MHCI
IO	0.49896671	0.18038488	1.38020314	0.18049481	14	12	riaz	MHCI
IO	0.47893362	0.36333878	0.63130453	1.75E-07	227	227	tcga	Checkpoint molecules
IO	0.53047203	0.2466364	1.14095314	0.10469667	20	20	allen	Checkpoint molecules
IO	0.59325921	0.28020074	1.25608698	0.1724949	15	27	liu	Checkpoint molecules
IO	0.95437163	0.35416572	2.57174863	0.92642876	15	11	riaz	Checkpoint molecules
IO	0.42085914	0.31750045	0.55786507	1.76E-09	227	227	tcga	NK cells
IO	0.43066657	0.19920376	0.93107526	0.03223313	18	22	allen	NK cells
IO	0.65372763	0.31520277	1.35582506	0.25342494	17	25	liu	NK cells
IO	0.51602203	0.17717994	1.5028718	0.22511322	15	11	riaz	NK cells
IO	1.75409344	1.33328858	2.30771031	5.94E-05	227	227	tcga	Matrix remodeling
IO	3.65286883	1.63044957	8.18390885	0.0016453	20	20	allen	Matrix remodeling
IO	1.46327324	0.66782985	3.20615884	0.34150812	27	15	liu	Matrix remodeling
IO	1.8890186	0.68270093	5.22687326	0.22061269	12	14	riaz	Matrix remodeling
IO	1.94989612	1.4747894	2.57805954	2.78E-06	227	227	tcga	Angiogenesis
IO	1.93459675	0.90157541	4.15124961	0.09026745	21	19	allen	Angiogenesis
IO	1.86582008	0.892888	3.89890401	0.09718239	23	19	liu	Angiogenesis
IO	1.80465743	0.62593523	5.20307577	0.27449724	11	15	riaz	Angiogenesis
IO	1.97472489	1.49685053	2.60516219	1.48E-06	227	227	tcga	Cancer-associated fibroblasts

Population	hr	hr_lower95	hr_upper95	pval*	n_low	n_high	cohort	var
IO	1.98349616	0.91511362	4.29920057	0.08270364	19	21	allen	Cancer-associated fibroblasts
IO	1.38564747	0.64212881	2.99008377	0.40588084	28	14	liu	Cancer-associated fibroblasts
IO	1.07221958	0.37089955	3.09963929	0.89755933	8	18	riaz	Cancer-associated fibroblasts
IO	1.87863847	1.4187695	2.48756582	1.07E-05	227	227	tcga	Endothelium
IO	1.01576509	0.46353656	2.22588421	0.96882738	24	16	allen	Endothelium
IO	0.87092976	0.41079463	1.84646682	0.71852204	24	18	liu	Endothelium
IO	1.9547557	0.70692985	5.40516122	0.19648771	12	14	riaz	Endothelium

*pval reported from a logrank statistical test

Supplementary Table 7. Pre-trained Clinical Transformer parameters. Column and row headers in bold.

	**GENIE**	**Chowell et al.**	**TCGA**	**Thorsson et al.**	**Oak trial**
**Feature types**	**Mutations, CNVs and demogra-phics**	**Aggregated molecular, clinical and demogra-phics**	**Immune related signatures**	**Immune signatures, molecular features and demogra-phics**	**molecular, demogra-phics**
**Epochs**	20000	30000	20000	20000	20000
**learning rate**	1.00E-04	1.00E-04	1.00E-04	1.00E-04	1.00E-04
**heads**	8	2	2	2	2
**layers**	8	2	8	8	8
**features percentile**	95	100	100	100	95
**Embeddings size**	128	128	128	128	128
**Parameters**	1M	202k	806k	801k	800k
**Estimated time**	100 hours	5 hours	15 hours	8 hours	3 hours
**Accelerator**	Tesla K80 11Gb	Tesla K80 11Gb	Tesla K80 11Gb	Tesla K80 11Gb	Tesla K80 11Gb
**Batch Size**	2048	1479	11069	6012	321
**Samples**	134626	1479	11069	6012	321
**Features (N)**	2285	17	29	49	414

Supplementary References

1. Hu, S., Fridgeirsson, E., van Wingen, G. & Welling, M. in Survival Prediction-Algorithms, Challenges and Applications 132-148 (PMLR, 2021).

2. Ching, T., Zhu, X. & Garmire, L.X. Cox-nnet: an artificial neural network method for prognosis prediction of high-throughput omics data. *PLoS computational biology* **14**, e1006076 (2018).

3. Katzman, J.L. et al. DeepSurv: personalized treatment recommender system using a Cox proportional hazards deep neural network. *BMC medical research methodology* **18**, 1-12 (2018).

4. Fotso, S. Deep neural networks for survival analysis based on a multi-task framework. *arXiv preprint arXiv:1801.05512* (2018).

5. Gensheimer, M.F. & Narasimhan, B. A scalable discrete-time survival model for neural networks. *PeerJ* **7**, e6257 (2019).

6. Chowell, D. et al. Improved prediction of immune checkpoint blockade efficacy across multiple cancer types. *Nature Biotechnology* **40**, 499-506 (2022).

7. Thorsson, V. et al. The immune landscape of cancer. *Immunity* **48**, 812-830. e814 (2018).

8. Samstein, R.M. et al. Tumor mutational load predicts survival after immunotherapy across multiple cancer types. *Nature genetics* **51**, 202-206 (2019).

9. Miao, D. et al. Genomic correlates of response to immune checkpoint blockade in microsatellite-stable solid tumors. *Nature genetics* **50**, 1271-1281 (2018).

10. Van Allen, E.M. et al. Genomic correlates of response to CTLA-4 blockade in metastatic melanoma. *Science* **350**, 207-211 (2015).

11. Bagaev, A. et al. Conserved pan-cancer microenvironment subtypes predict response to immunotherapy. *Cancer cell* **39**, 845-865. e847 (2021).

12. Liu, D. et al. Integrative molecular and clinical modeling of clinical outcomes to PD1 blockade in patients with metastatic melanoma. *Nature medicine* **25**, 1916-1927 (2019).

13. Riaz, N. et al. Tumor and microenvironment evolution during immunotherapy with nivolumab. *Cell* **171**, 934-949. e916 (2017).
