# Supplementary material for: Pretrained transformers applied to clinical studies improve predictions of treatment efficacy and associated biomarkers
Source: Nat Commun. 2025 Mar 1;16:2101. doi: 10.1038/s41467-025-57181-2 (PMC11873189; doi:10.1038/s41467-025-57181-2)
Supplement: Supplementary file 2 — Reporting Summary [file 41467_2025_57181_MOESM2_ESM.pdf]

nature portfolio

Corresponding author(s): Gustavo Arango-Argoty
Etai Jacob

Last updated by author(s): Oct 1, 2024

Reporting Summary

Nature Portfolio wishes to improve the reproducibility of the work that we publish. This form provides structure for consistency and transparency in reporting. For further information on Nature Portfolio policies, see our Editorial Policies and the Editorial Policy Checklist.

Statistics

For all statistical analyses, confirm that the following items are present in the figure legend, table legend, main text, or Methods section.

n/a	Confirmed	
☐	☒	The exact sample size (*n*) for each experimental group/condition, given as a discrete number and unit of measurement
☒	☐	A statement on whether measurements were taken from distinct samples or whether the same sample was measured repeatedly
☐	☒	The statistical test(s) used AND whether they are one- or two-sided<br>*Only common tests should be described solely by name; describe more complex techniques in the Methods section.*
☐	☒	A description of all covariates tested
☐	☒	A description of any assumptions or corrections, such as tests of normality and adjustment for multiple comparisons
☐	☒	A full description of the statistical parameters including central tendency (e.g. means) or other basic estimates (e.g. regression coefficient) AND variation (e.g. standard deviation) or associated estimates of uncertainty (e.g. confidence intervals)
☐	☒	For null hypothesis testing, the test statistic (e.g. *F*, *t*, *r*) with confidence intervals, effect sizes, degrees of freedom and *P* value noted<br>*Give P values as exact values whenever suitable.*
☒	☐	For Bayesian analysis, information on the choice of priors and Markov chain Monte Carlo settings
☒	☐	For hierarchical and complex designs, identification of the appropriate level for tests and full reporting of outcomes
☒	☐	Estimates of effect sizes (e.g. Cohen's *d*, Pearson's *r*), indicating how they were calculated

Our web collection on statistics for biologists contains articles on many of the points above.

Software and code

Policy information about availability of computer code

Data collection	No data was collected for this study
Data analysis	Code for the Clinical Transformer and to reproduce the analyses in this manuscript is publicly available on Github: https://github.com/gaarangoa/ClinicalTransformer<br>flaml==1.0.11, lifelines==0.27.1, numpy==1.22.4, pandas==1.4.3, python==3.8.10, scikit-learn==0.24.1, scipy==1.9.0, tensorflow==2.10.0rc1, umap-learn==0.5.3

For manuscripts utilizing custom algorithms or software that are central to the research but not yet described in published literature, software must be made available to editors and reviewers. We strongly encourage code deposition in a community repository (e.g. GitHub). See the Nature Portfolio guidelines for submitting code & software for further information.

Data

Policy information about availability of data

All manuscripts must include a data availability statement. This statement should provide the following information, where applicable:

- Accession codes, unique identifiers, or web links for publicly available datasets
- A description of any restrictions on data availability
- For clinical datasets or third party data, please ensure that the statement adheres to our policy

All datasets (except for the MYSTIC clinical trial) are publicly available at the sources listed in Table 1. For MYSTIC, the AstraZeneca group of companies allows researchers to submit a request to access anonymized patient-level clinical data, aggregated clinical or genomics data (when available), and/or anonymized clinical study documents through the Vivli (https://vivli.org) web-based data request platform, in accordance with AstraZeneca's clinical data access policy: https://www.astrazenecaclinicaltrials.com/our-transparency-commitments

Research involving human participants, their data, or biological material

Policy information about studies with human participants or human data. See also policy information about sex, gender (identity/presentation), and sexual orientation and race, ethnicity and racism.

Reporting on sex and gender	NA
Reporting on race, ethnicity, or other socially relevant groupings	NA
Population characteristics	NA
Recruitment	NA
Ethics oversight	NA

Note that full information on the approval of the study protocol must also be provided in the manuscript.

Field-specific reporting

Please select the one below that is the best fit for your research. If you are not sure, read the appropriate sections before making your selection.

☒ Life sciences ☐ Behavioural & social sciences ☐ Ecological, evolutionary & environmental sciences

For a reference copy of the document with all sections, see nature.com/documents/nr-reporting-summary-flat.pdf

Life sciences study design

All studies must disclose on these points even when the disclosure is negative.

Sample size	NA, study is retrospective in nature and sample sizes were dictated by data available for retrospective analysis. No sample size calculation performed.
Data exclusions	For MYSTIC clinical trial, patients were only included if they (1) received immunotherapy and (2) had tissue tumor mutation burden data available. Rationale: analysis required immunotherapy patients and a complete set of features.<br><br>For public datasets, no exclusions were made except for the Riaz et al., 2017 and Liu et al., 2019 datasets, where patients were included only if they received prior immunotherapy and had whole transcriptome data from samples collected prior to immunotherapy. Rationale: analysis required baseline samples and a comparable cohort across independent studies.
Replication	Repeated k-fold cross validation was performed within given datasets; in addition, we evaluated our findings over multiple independent test datasets
Randomization	NA, study is retrospective in nature and groups were dictated by data available for retrospective analysis.
Blinding	NA, study is retrospective in nature and groups were dictated by data available for retrospective analysis.

Reporting for specific materials, systems and methods

We require information from authors about some types of materials, experimental systems and methods used in many studies. Here, indicate whether each material, system or method listed is relevant to your study. If you are not sure if a list item applies to your research, read the appropriate section before selecting a response.

Materials & experimental systems

n/a	Involved in the study
☒	☐ Antibodies
☒	☐ Eukaryotic cell lines
☒	☐ Palaeontology and archaeology
☒	☐ Animals and other organisms
☐	☒ Clinical data
☒	☐ Dual use research of concern
☒	☐ Plants

Methods

n/a	Involved in the study
☒	☐ ChIP-seq
☒	☐ Flow cytometry
☒	☐ MRI-based neuroimaging

Clinical data

Policy information about clinical studies

All manuscripts should comply with the ICMJEguidelines for publication of clinical research and a completedCONSORT checklist must be included with all submissions.

Clinical trial registration	NCT02453282 (MYSTIC clinical trial)
Study protocol	doi: 0.1001/jamaoncol.2020.0237, supplement 2. All protocols and amendments were approved by the Institutional Review Boards or Ethics Committees of participating centers.
Data collection	doi: 0.1001/jamaoncol.2020.0237, methods: study was performed at 203 cancer treatment centers in 17 countries. Data were collected from July 21, 2015 to October 30, 2018. Study was performed in accordance with the Declaration of Helsinki and the International Conference on Harmonization Good Clinical Practice guidelines. All patients provided written informed consent.
Outcomes	NA, study is retrospective in nature and clinical trial endpoints were not evaluated or considered

Plants

Seed stocks	*Report on the source of all seed stocks or other plant material used. If applicable, state the seed stock centre and catalogue number. If plant specimens were collected from the field, describe the collection location, date and sampling procedures.*
Novel plant genotypes	*Describe the methods by which all novel plant genotypes were produced. This includes those generated by transgenic approaches, gene editing, chemical/radiation-based mutagenesis and hybridization. For transgenic lines, describe the transformation method, the number of independent lines analyzed and the generation upon which experiments were performed. For gene-edited lines, describe the editor used, the endogenous sequence targeted for editing, the targeting guide RNA sequence (if applicable) and how the editor was applied.*
Authentication	*Describe any authentication procedures for each seed stock used or novel genotype generated. Describe any experiments used to assess the effect of a mutation and, where applicable, how potential secondary effects (e.g. second site T-DNA insertions, mosiacism, off-target gene editing) were examined.*
